# Nitrogen topdressing timing modifies free amino acids profiles and storage protein gene expression in wheat grain

**DOI:** 10.1186/s12870-018-1563-3

**Published:** 2018-12-13

**Authors:** Yingxin Zhong, Dachao Xu, Kim Henrik Hebelstrup, Donglei Yang, Jian Cai, Xiao Wang, Qin Zhou, Weixing Cao, Tingbo Dai, Dong Jiang

**Affiliations:** 1 National Technique Innovation Center for Regional Wheat Production / Key Laboratory of Crop Physiology and Ecology in Southern China, Ministry of Agriculture / National Engineering and technology Center for Information Agriculture, Nanjing Agricultural University, Nanjing, People’s Republic of China; 2 National Laboratory of Plant Genetics and Germplasm Enhancement, Nanjing Agricultural University, Nanjing, People’s Republic of China; 3 Department of Molecular Biology and Genetics, Section of Crop Genetics and Biotechnology, Aarhus University, Flakkebjerg, Forsøgsvej 1, 4200 Slagelse, Denmark

**Keywords:** Amino acid, Glutenin subunit, Nitrogen topdressing timing, Quaulity, Wheat

## Abstract

**Background:**

Nitrogen is one basic element of amino acids and grain protein in wheat. In field experiments, wheat plants were subjected to different timing of nitrogen topdressing treatments: at the stages of emergence of the top fifth leaf (TL5), top third leaf (TL3) and top first leaf (TL1) to test the regulatory effects of nitrogen topdressing timing on grain protein quality. The underlying mechanisms were elucidated by clarifying the relationship between proteolysis in vegetative organs and accumulation of amino acids in the endosperm cavity, conversion of amino acids, and storage protein synthesis in endosperm of wheat grain.

**Results:**

Delayed nitrogen topdressing up-regulated gene expression related to nitrogen metabolism and protease synthesis in the flag leaf, followed by more free amino acids being transported to both the cavity and the endosperm from 7 days after anthesis (DAA) to 13 DAA in TL1. TL1 enhanced the conversion between free amino acids in endosperm and upregulated the expression of genes encoding high molecular weight (HMW) and low molecular weight (LMW) subunits and protein disulfide isomerases-like (PDIL) proteins, indicating that the synthesis and folding of glutenin were enhanched by delayed nitrogen topdressing. As a consequense, the content of glutenin macropolymers (GMP) and glutenin increased with delaying nitrogen topdressing.

**Conclusions:**

The results highlight the relationship between nitrogen remobilization and final grain protein production and suggest that the nitrogen remobilization processes could be a potential target for improving the quality of wheat grain. Additionally, specific gene expression related to nitrogen topdressing was identified, which conferred more detailed insights into underlying mechanism on the modification protein quality.

**Electronic supplementary material:**

The online version of this article (10.1186/s12870-018-1563-3) contains supplementary material, which is available to authorized users.

## Background

Wheat is one of the most important staple crops, providing more than 20% of the daily dietary protein for human globally [1]. Global demand of wheat has been increasing in the last few decades, with an average global energy supply increase from 441 kcal capita^− 1^ day^− 1^ in 1963 to 552 kcal capita^− 1^ day^− 1^ in 2013 (http://www.fao.org/faostat/zh/#data/FBS). In China, wheat and its products account for 17.5% of the total daily intake of energy and 17.7% of protein (http://www.fao.org/faostat/zh/#data/FBS).

Wheat flour has a distinctive nature for baking properties due to its unique gluten proteins as compared to other cereals. Gluten contains hundreds of different proteins that are either monomers (gliadins) or polymers (glutenins) [2]. The gliadins mainly form intramolecular disulfide bonds, which make them less important than glutenins for bread-baking [3]. The glutenin polypeptides form large polymers via intermolecular disulfide bonds, which confer the dough elasticity (strength). Glutenin macropolymers (GMP) is one of the large type glutenin polymers insoluble in sodium dodecyl sulfate (SDS) [4], which is closely correlated to the baking performances of wheat flour. After being separated in SDS-PAGE, GMP is further divided into the high molecular weight (HMW-) and low molecular weight (LMW-) subunits [5]. Therefore, the formation of the above-mentioned storage proteins in wheat endosperm is crucial for the final bread-making quality.

Limitations of protein deposition in wheat grain involve a balance between the capacity of substrate production in plant (source-limited), and utilization capacity of the substrate in grains (sink-limited) [6]. Though some sink-related factors such as starch accumulation and grain size affect the biosynthesis of seed storage proteins, the source-limitation is reported to depress storage protein deposit in a large extent [6]. Namely, protein synthesis in grain is largely dependent on the supply of amino acid precursors being transported into the endosperm from the sources elsewhere in the plants [7]. These precursors come from the hydrolysis of large protein molecules into amino acids by proteolytic enzymes in vegetative organs [8]. It has been shown that there is a parallel change in the content and composition of amino acids in the flag leaf, phloem exudate and the sap of the endosperm cavity and of the whole endosperm [9]. Barneix et al. (2007) reported that the final grain protein content (GPC) is correlated with the content of free amino acids in flag leaves during grain filling [10]. It may therefore be possible to increase the amount of grain protein if more available amino acids are produced by protein hydrolysis in vegetative organs (such as green leaves) during the late grain filling stage.

Wheat grain protein content is dependent on genetic, environmental and management factors as well as the interaction between these factors [11]. It is generally accepted that nitrogen (N) fertilization is the most effective management practice to regulate grain protein content [12]. N is usually split fertilized into doses of the basal N applied before sowing and the topdressing N applied during the growth period (mostly at the jointing stage, and sometimes further split at both jointing and booting stage) to produce both high grain yield and protein [13]. N topdressed at the early to mid-vegetative growth stages primarily increases yield potential rather than grain protein content [13], whereas N applied during reproductive development stages enhances protein synthesis in grains [14]. For instance, splitting the use of N at booting or a later growth stage is found to enhance grain protein content and bread loaf volume [15]. N application at spike formation stage increased gluten strength of cultivars with relatively short maturation period under a cool climate [16, 17]. However, Johansson et al. (2004) reported that late N application significantly decreased gluten strength rather than protein concentration [18]. Other studies have also shown that delaying N fertilization restricts the conversion of N into storage proteins [13]. However, there is a lack of comprehensive studies that describes regulatory effects of N topdressing timing on: production of amino acids by proteolysis in leaves, the consequent contribution to amino acids in the endosperm cavity sap, and the conversion of the amino acids for synthesis of storage protein in wheat endosperm.

Therefore, based on a field experiment, we report the effects of N topdressed at different growth stages indicated by the leaf age on protein deposit in wheat grain. The time-course dynamic changes in the expression of genes involved in proteolysis in flag leaves during grain filling were investigated. Changes in contents of free amino acids in both the endosperm cavity sap and the developing endosperms during grain filling were followed. The conversion of amino acids in grains and its contribution to synthesis of storage proteins were also studied. The aims of the present study were (1) to clarify the relationship between proteolysis in vegetative organs and accumulation of amino acids in the endosperm cavity, conversion of amino acids and storage protein synthesis in endosperm of wheat grain; (2) to prove the regulatory effects of N topdressing timing on grain protein quality and (3) to elucidate the underlying mechanisms in wheat.

## Methods

### Plant materials and growth conditions

The experiment was conducted at the Tangquan Experimental Station of Nanjing Agricultural University, Nanjing (32°08′ N and 118°51′ E), Jiangsu Province, P. R. China in the growth season of 2014–2015. The soil is a clay containing 15.7 g kg^− 1^ organic matter, 57.7 mg kg^− 1^ available N, 1.51 g kg^− 1^ total N, 288.1 mg kg^− 1^ available K and 40.3 mg kg^− 1^ Olsen P, with pH of 7.8. Yangmai 16, a locally released and widely planted winter wheat (*Triticum aestivum* L.) cultivar was used. The sowing density was 180 seedlings m^− 2^ with a row space of 25 cm. The total N rate was 240 kg ha^− 1^ of which half was applied before sowing, while the rest share was topdressed basing at the leaf age as described below. Overall, three N topdressing timings were included: at the emergence of the top-fifth-leaf (TL5), the top-third-leaf (TL3, in accordance to the jointing stage, which is the recommendation timing of nitrogen topdressing in practice), and at the top-first-leaf (the emergence of the flag leaf, TL1) of the main stem, respectively. The plot size was 3 m × 3.2 m. The experiment was a randomized complete block design, with three biological replicates for each treatment.

### Sampling and harvest

Uniform stems flowering on the same day were tagged. The flag leaf and grains attach to the tagged stems were separately collected at 7, 13, 19, 25 and 31 days after anthesis (DAA). One batch of the leaf samples was killed at 105 °C for 1 h and then dried to constant weight at 80 °C in an oven, while another batch of leaves was immediately put into liquid N and then stored at − 80 °C. Grains from the central spikelets of the ears were divided into two batches as well. One batch was used for freeze-stored samples as mentioned above, and another batch was used for collection of sap from the endosperm cavity by a capillary method (described below) immediately after sampling. The dry samples of grain and leaf were milled into powder and stored at 4 °C until analysis, and the freeze-treated grain and leaf samples were used for analysis of gene expression and contents of amino acids.

At maturity, heads of 4 m^2^ were harvested to get grain samples. Grains were dried and kept for at least two months before milling with a Buhler experimental mill (MLU-202, Buhler Equipment Engineering Wuxi Co., Ltd., China).

### Identification of seed proteins

The protein fractions were separated and analyzed according to the protocol reported by Luthe (1983) [19]. Four protein fractions were sequentially extracted in the order given below by stirring 500 mg flour in 25 ml given extraction solvents for 2 h at room temperature as: albumin in 10 mM Tris–HCl (pH 7.5), globulin in 1 M NaCl with 10 mM Tris–HCl (pH 7.5), gliadin in 70% (v/v) ethanol with 10 mM Tris–HCl (pH 7.5), and glutenin in 0.5% SDS and 1% β-mercaptoethanol with 10 mM Tris-HCl (pH 7.5). The contents of protein and protein fractions (glutenin and gliadin fraction) in flour were determined by the micro-Kjeldahl method of AACC 46–13.01 [20].

Fifty mg flour from each layer were suspended in 1 ml of SDS (1.5%) solution and then centrifuged at 15500 *g* at 20 °C for 30 min. The sediment was washed twice with SDS solution (1.5%). Then the sediment was then dissolved in 2 ml NaOH (0.2%) for 30 min. Afterwards, 3 ml Biuret reagent was added to the solution to evaluate the N concentration for further calculation of GMP content [21].

Total HMW-GS and LMW-GS were separated using our previous method [22, 23]. In short, 80 mg grain meal was dissolved in 1 ml isopropyl alcohol (50%), then water bathed at 65 °C for 20 min. The mixture was then centrifuged at 9200 *g* for 5 min. The sediment was kept and the supernatant containing gliadins was discarded. Thereafter, 100 μl extraction buffer A containing 2% Dithiothreitol, 40 mM Tris-HCl (pH 8.0), 2% SDS, 25% isopropyl alcohol was added to sediment. The mixture was further water bathed at 65 °C for 30 min to reduce disulfide bonds of proteins. Afterwards, 100 μl extraction buffer B containing 1.4% 4-vinylpyridine, 40 mM Tris-HCl (pH 8.0), 2% SDS, 25% isopropyl alcohol was added to the mixture, and then water bathed at 65 °C for 15 min to allow better separation of proteins by alkylation. After centrifugation at 9200 *g* for 5 min, the supernatant containing proteins was collected and then put in a new centrifuge tube, to which 100 μl glutenin extraction buffer containing 62.5 mM Tris-HCl (pH 6.8), 0.2% SDS, 5% β-mercaptoethanol, 40% sucrose, 0.5% bromophenol blue was added. The mixture was water bathed at 100 °C for 5 min followed by centrifugation at 9200 *g* for 5 min. The supernatant was used for SDS-PAGE.

The SDS-PAGE was carried out with our previous protocol [23]. Quantifications of HMW-GS and LMW-GS were conducted by analysis software QUANTITY ONE. During the quantification procedure, a standard protein (Cat NO. 1610373, Bio-Rad, USA) with given concentration was separately loaded in 5, 10 and 15 μl volumes to three lanes on the same gel. Standard proteins were used to make a standard curve. The content of each HMW-GS and LMW-GS in each lane was then quantified.

### Contents of amino acids in grain and endosperm cavity

The grain powder samples were soaked by 0.1 M HCl for 24 h. After filtration, the filtrate was added with three volumes of 10% sulfosalicylic acid, and the resulting solution was filtered again for analysis of amino acids with an L-8900 High Speed Amino Acid Analyzer (Hitachi Corp., Japan). Content of total amino acids (free amino acids and protein amino acids) was measured by hydrolysis of powder samples with 6 M HCl by the same analyzer. The contents of each amino acid and total free amino acids in grain was expressed on a dry weight base.

Concentration of individual free amino acid in the sap of endosperm cavity was only determined between 7 DAA and 25 DAA, since at 31 DAA the the sap was too little to be collected. The sap was collected by slicing off the hilum end of the grain, sucking the fluid with a capillary and pooling the fluid of thirty grains for each replicate. After freeze-drying, the content of free amino acids was analyzed as method above. The content of free amino acids in endosperm sap was expressed as arbitrary units because of the difficulty to accurately measure the volume of the sap. For each sample (replicate), the assay was done three times. During the acid hydrolysis procedure, Asn and Gln were deaminated to Asp and Glu, respectively, and Trp was also completely destroyed. Thus, contents of only the remaining 17 kinds of amino acids are given.

### Analysis of qRT-PCR

Developing seeds from the central spikelets of five spikes were combined for extraction of total RNA by using the Total RNA Purification Kit (Genemark) according to the manufacturer’s instructions. Approximately 500 ng purified mRNA was used to synthesize cDNA by using a HiScript II One Step RT-PCR Kit (Vazyme, China). The cDNA was used for quantitative real-time PCR (qRT-PCR). Specific primers separately for three pairs of LMW-GS specific primers (specific for LMW-GS genes located on A, B and D chromosome, respectively), two pairs of protease genes specific primers and six pairs of amino acid metabolism genes were designed to measure the gene expression levels. The other primers were similar to those used in previous work [24, 25]. ADP-ribosylation factor (ADP-RF) was used as housekeeping gene as described previously [26]. The functions of genes analysed in this study are listed in Additional file 1: Table S1. Details of the primers are listed in Additional file 1: Table S2. All primers were gene specific, as checked by electrophoresis and unique melting peak verification. The efficiencies of all primers ranged from 90 to 100%, which was determined by standard curves using a series of cDNA dilutions at 13 DAA in TL3. The qPCR reactions included an initial incubation at 94 °C for 3 min, followed by 40 cycles at 94 °C for 20 s, 58 °C for 15 s, 72 °C for 20 s. The gene expression data were standardized according to the CFX96 Real-Time system (Bio-Rad). Triplicate for each PCR reaction and three biological replicates were performed for each gene.

### Statistics

All data were subjected to analysis of variance (ANOVA) using the SPSS (Statistical Product and Service Solutions) Version 17.0. The ANOVA mean comparisons were performed in terms of the least significant difference (LSD), at the significant level of *P* < 0.05.

## Results

N contents in the vegetative organs of the main stem (leaf + stem + glume) at anthesis were 40.4, 48.1, 51.5 mg per stem in TL5, TL3 and TL1, respectively (Additional file 1: Table S3), while the N contents (leaf + stem + glume) at maturity dropped to only 5.5, 3.6, 4.6 mg per stem in TL5, TL3 and TL1, respectively. Correspondingly, N contents in mature grain reached 55.57, 67.23 and 66.2 mg per stem in TL5, TL3 and TL1, respectively. Thus, a high share of the N that accumulated in the vegetative organs before anthesis was redistributed into grains during grain filling. More than 60% of N accumulated before anthesis was transferred into grains in all three top-dressing trials, however the amount was lowest in TL5 and highest in TL1. This indicates that delaying topdressing N enhances the remobilization of the pre-anthesis accumulated N in vegetative organs, and increases its contribution to N content during grain filling.

### N assimilation and proteolysis of mobilizable proteins in flag leaves

**Fig. 1:**
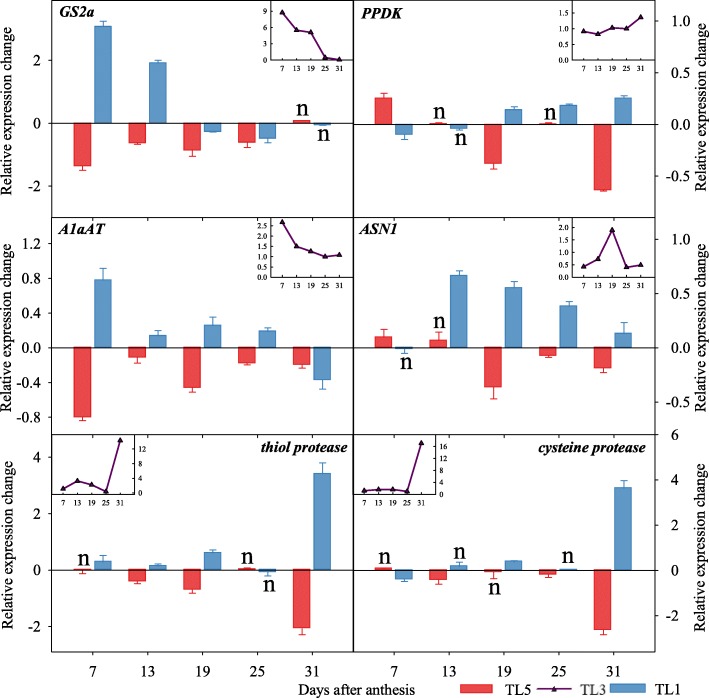
Expression levels of genes involving in nitrogen metabolisms and protein degradation and remobilization in wheat leaves. Note: TL5, TL3 and TL1 indicate topdressing timing of nitrogen at the growth stage of fifth, third and first leaf (flag leaf) from the top, respectively. The line charts represent gene expression during grain filling stage in TL3. The bar charts represent the relative variation of TL5 (red) and TL1 (blue) in relation to TL3. Bars indicate standard errors. Function of each gene is explained in Additional file 1: Table S1

Glutamine synthetase (GS), cytosolic pyruvate orthophosphate dikinase (PPDK), alanine aminotransferase (AlaAT), asparagine synthetase (AS), thiol- and cysteine proteases are key enzymes involving in processes of synthesis and degradation of proteins in plants (see details in the discussion session). Here, we analyzed the expressions of the corresponding genes *GS2a, PPDK, AlaAT* and *ASN1* in flag leaves during the time of grain filling (Fig. 1). The expression levels of *GS2a* and *AlaAT* dropped sharply from 7 DAA (days after anthesis) to 13 DAA. Almost no expression of *GS2a* was detected since at 25 and 31 DAA, while expression of *AlaAT* was lower at 19 DAA and onwards. The expression of *PPDK* slightly increased from 13 DAA to 25 DAA, and increased further at 31 DAA. The expression of *ASN1* increased from 13 DAA and peaked at 19 DAA, and dropped rapidly thereafter. The expressions of *thiol*- and *cysteine*-*proteases* remained stable between 7 DAA and 25 DAA, and jumped rapidly from 25 DAA to 31 DAA, where it was 4–5 fold higher than that of the early filling stages.

To better illustrate the effect of N topdressing timing on synthesis and degradation of proteins in leaves, TL3 (the jointing stage, which is the recommendation N topdressing stage in practice) was taken as the control, the relative variations of TL5 and TL1 in relation to TL3 were then calculated (Fig. 1). The bars above and below the x-axis indicate upregulation and downregulation of the given gene, respectively. In comparison to TL3, *GS2a* in TL1 was significantly upregulated at 7 DAA and 13 DAA, while it was close to TL3 between 19 DAA and 31 DAA. *GS2a* was significantly lower from 7 DAA to 25 DAA in TL5 than TL3. Similarly, the expression of *A1aAT* was higher in TL1 while lower in TL5 from 7 DAA to 25 DAA. The expression of *PPDK* was higher at 7 DAA and lower at and after 19 DAA in TL5, while the trend was reversed in TL1 in comparison to TL3. The expression of *ASN1* was close among the three treatments at 7 DAA, while it was higher in TL1 from 13 DAA to 31 DAA and lower in TL5 from 19 DAA to 31 DAA. The expression levels of the two *proteases* at 31 DAA were much higher in TL1 while it was much lower in TL5 in relation to TL3.

### Contents of amino acids in sap of endosperm cavity

**Fig. 2:**
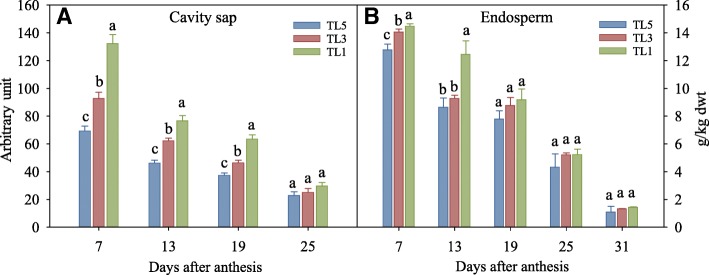
Content of total free amino acids in sap of endosperm cavity (**a**) and in the endosperm (**b**) of wheat grain. Note: TL5, TL3 and TL1 indicate topdressing timing of nitrogen at the growth stage of fifth, third and first leaf (flag leaf) from the top, respectively. Bars indicate standard errors. The complete data of content of free amino acid content are listed in Additional file 1: Table S4–1 (sap of cavity) and Additional file 1: Table S4–2 (endosperm)

Contents of individual and total free amino acids in the sap of the cavity during grain filling are shown in Table S4–1 and Fig. 2a. Here, the content of total free amino acids in the cavity sap was highest at 7 DAA, followed by a rapid drop between 7 DAA and 13 DAA, and then continued to decrease until 25 DAA. The content of total free amino acids in the cavity sap was higher in TL1 while it was lower in TL5 during grain filling as compared with TL3, especially at the earlier grain filling period. For instance, the level of total free amino acids content was separately 42.6 and 25.4% higher at 7 DAA and 19 DAA in TL1 in comparison to TL3, while the level of total free amino acids content was separately 25.2 and 9.2% lower at 7 DAA and 25 DAA in TL5 in comparison to TL3. Similarly, the content of Glu, which accounts for approximate 40% of total free amino acids, dropped continuously from 7 to 25 DAA. In relation to TL3, TL1 increased 25.0 and 30.0% of glutamine content, while TL5 decreased 29.1 and 35.0% at 7 and 13 DAA, respectively. However, there was no significant difference in glutamine content between treatments at 25 DAA. In summary, we therefore saw that delaying N topdressing timing enhanced accumulation of free amino acids in the wheat endosperm cavity, while the opposite was the case for early N application.

### Contents and conversion of amino acids in endosperm

Content of total free amino acids in the endosperm decreased along with grain filling. The content dropped 14 fold and 31 fold from 7 DAA to 31 DAA in TL5 and TL1, respectively (Fig. 2b). In general, early N topdressing gave a lower content of total free amino acids in endosperm, while the opposite is the case when delaying N application, especially for the early grain filling stages. Here, in relation to TL3, the content of total free amino acids was increased by 34.2% at 13 DAA in TL1, while it was decreased by 9.2% at 7 DAA in TL5 compared with TL3. However, the differences were not significant between the treatments after 19 DAA.

**Fig. 3:**
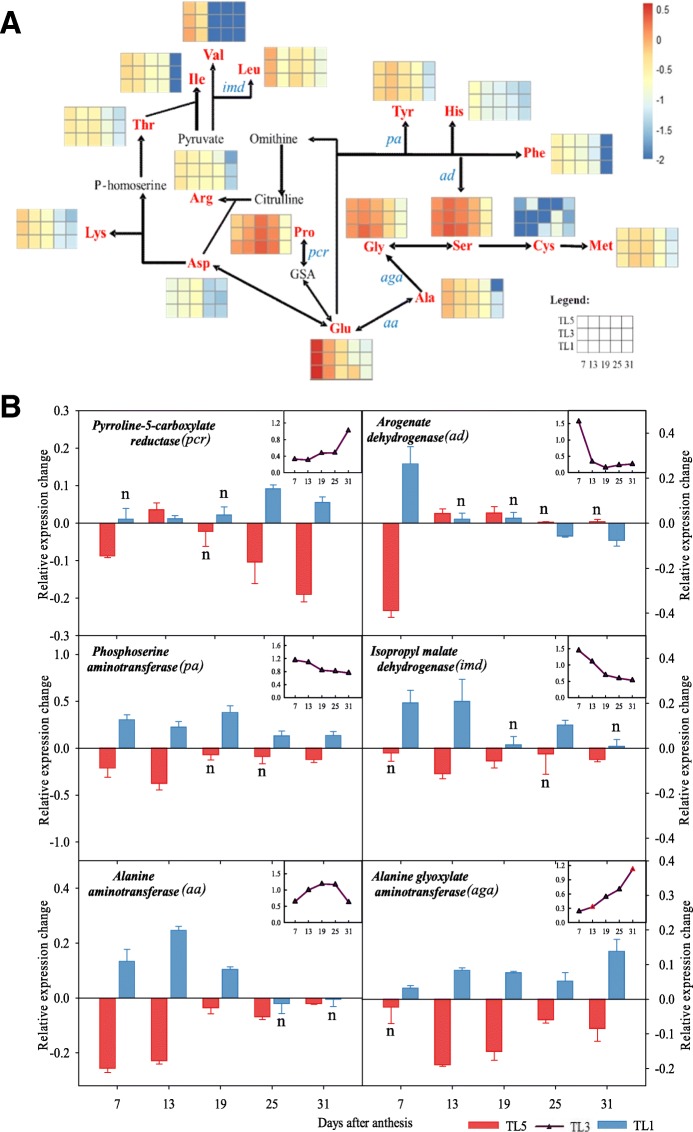
Heatmap of changes in content of free amino acids (**a**) and expression levels of representative genes involving in metabolism of amino acids (**b**) in wheat endosperm as affected by nitrogen topdressing timing. Note: A. TL5, TL3 and TL1 indicate topdressing timing of nitrogen at the growth stage of fifth, third and first leaf (flag leaf) from the top, respectively. Content of each amino acid was the mean of three biological replicates. The content was normalized after logarithm of each free amino acid is shown in reference to the continuous scale color bar. The complete data of content of free amino acids are shown in Additional file 1: Table S1–2. FAA, free amino acid. GSA indicates c-glutamyl semialdehyde, and P-homoserine indicates phosphohomoserine. *Pcr, pyrroline-5-carboxylate reductase; pa, phosphoserine aminotransferase; ad, arogenate dehydrogenase; imd, isopropyl malate dehydrogenase; aa, alanine aminotransferase; aga, alanine glyoxylate aminotransferase.* B. The line charts represent genes expression during grain filling stage in TL3. The bar charts represent the relative variation of TL5 (red) and TL1 (blue) in relation to TL3. All data were subjected to one-way analysis of variance (ANOVA) to determine the significant differences between treatments. Bars indicate standard errors. n, not significant (*P* > 0.05). The amino acids metabolic process that each gene involved in are shown as abbreviations in blue in Fig. 3a

Figure 3a shows a heatmap of individual free amino acids in the endosperm during the filling stage. The figures are shown as normalized data rather than specific concentrations to focus on changes of individual amino acids during the filling stage as well as differences between different treatments, which would otherwise be masked by the large amount of Glu. The pathways of amino acid metabolism are displayed as the major pathway groups as described by Singh (Singh 1998). In endosperm, Glu is also the major component of the free amino acids pool (Fig. 3a), together with serine (Ser), glycine (Gly), lysine (Lys) and leucine (Leu), of which each accounts for more than > 6% of the total pool at 7 DAA (Additional file 1: Table S4–2). Glu content fell rapidly during grain filling, while the contents of Ser, Gly and Tyr increased between 7 DAA to 13 DAA, Pro increased between 7 to 19 DAA and Leu increased between 13 to 19 DAA (Fig. 3a). Thereafter, content of each type of free amino acid decreased continuously after 13 DAA and very little free amino acid was detected at 31 DAA in the endosperm.

In accordance with the observed effect of N topdressing timing on total free amino acid content, significant differences between treatments were observed for some of the free amino acids between 7 to 25 DAA. For instance, delayed N topdressing (TL1) increased the content of Glu (7 to 13 DAA), Ser and Gly (13 to 19 DAA), Pro (7 to 25 DAA) and Val (7 DAA). Some less abundant amino acids were also increased in TL1 such as Arg (7 DAA) and Met (13 DAA). Early N topdressing (TL5) decreased the content of Glu, Ser and Val (7 DAA), Pro (13 to 25 DAA) and Leu (19 DAA). However, the difference between treatments disappeared at the end of filling stage.

Expression of six representative genes involved in the amino acid metabolism pathway in endosperm during filling stage were assayed. The expression of *pyrroline-5-carboxylate reductase (pcr)* and *alanine glyoxylate aminotransferase (aga)* continuously increased over grain filling in all treatments (Fig. 3b). In contrast, the expression of *arogenate dehydrogenase* (ad) was very high at the earlier grain filling stage, it declined rapidly from 7 DAA to 13 DAA, and then maintained at very low level between 19 DAA and 31 DAA. Similarly, there was a slight decrease from 7 DAA to 19 DAA in the expression of *phosphoserine aminotransferase (pa)* and *isopropyl malate dehydrogenase (imd)*, and then it remained at a relatively stable level after 19 DAA. The expression of *alanine aminotransferase (aa)* increased from 7 DAA to a peak at 19 DAA or 25 DAA, and then decreased thereafter.

In general, early N topdressing (TL5) down-regulated the expressions of the above-mentioned genes. In particular, expression levels of *pcr* at late grain filling, of *ad, imd* and *aa* at early grain filling, and of *aga* from 13 DAA to 31 DAA were lower in TL5 than in TL3, while it was the opposite for TL1. Moreover, the expressions of *pa* over the whole filling stage were higher in TL1 while lower in TL5 as compared with TL3.

### Synthesis and aggregation of glutenin subunits in endosperm

**Fig. 4:**
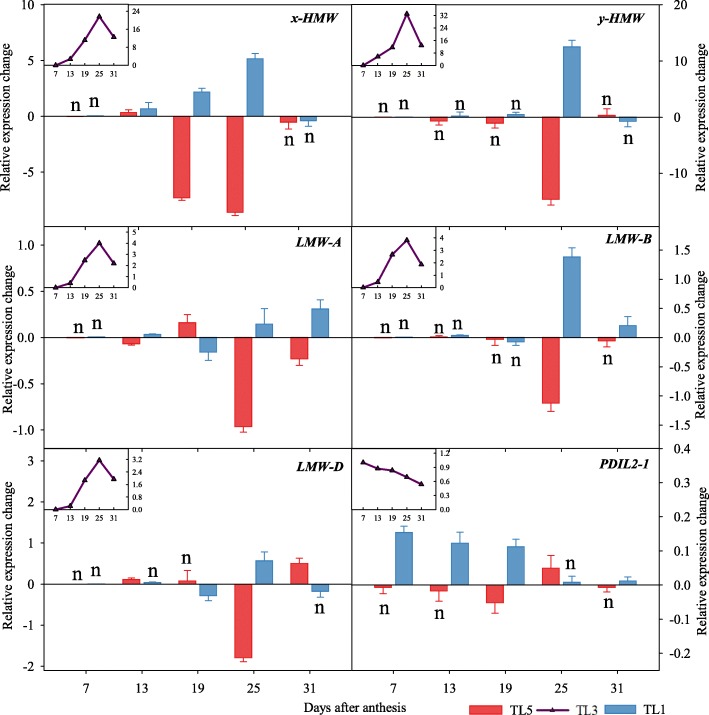
Expression levels of genes involving in glutenin synthesis and formation of glutenin polymers in wheat grains. Note: TL5, TL3 and TL1 indicate topdressing timing of nitrogen at the growth stage of fifth, third and first leaf (flag leaf) from the top, respectively. The line charts represent genes expression during grain filling stage in TL3. The bar charts represent the relative variation of TL5 (red) and TL1 (blue) in relation to TL3. Bars indicate standard errors. Function of each gene is explained in Additional file 1: Table S1

Expression levels of genes encoding the HMW-GS and LMW-GS were analyzed. Genes encoding both HMW-GS and LMW-GS were expressed at the highest at 25 DAA (Fig. 4). There was no significant difference among the N topdressing timing treatments in the expression of genes encoding the x-type and y-type HMW-GS at 7 DAA, 13 DAA and 31 DAA. However, late topdressing (TL1) showed much higher expression of both x-type and y-type HMW-GS at 25 DAA, while it was the opposite for TL5. The expressions levels of genes encoding LMW-GS located at *1A* were not significantly different between TL3 and TL1 at 25 DAA, while they were lower in TL5 than TL3 and TL1. The expressions of genes encoding LMW-GS located at *1B* and *1D* in response to N topdressing timing were similar to those encoding HMW-GS. *PDIL2–1*, encoding the protein being responsible for assisting disulfide bond formation as well as correcting folding of the storage proteins, showed a decreased expression during grain filling. The expression of *PDIL2–1* was higher in TL1 than in other treatments from 7 DAA to 19 DAA.

### Grain protein quality modified by N topdressing timing

Significant effects of N topdressing timing were found on the contents of protein, gliadin, glutenin and glutenin subunits in mature grains (Table 1). The late N application (TL1) increased protein content by 9.8% in relation to TL3. Reversely, predating topdressed N (TL5) caused reduction of protein content by 3.6%. Contents of the gliadin and glutenin in flour showed the same trends as observed for total protein. In relation to TL3, TL1 increased gliadin and glutenin content by 24.0 and 23.8%, respectively. Whereas TL5 resulted in decrements of gliadin and glutenin by 12.5 and 16.8% in comparison to TL3. The contents of the GMP and its two major components (HMW-GS and LMW-GS) were also affected by N topdressing timing, generally increased by delaying N topdressing timing.

**Table 1:** Effects of nitrogen topdressing timing on contents of total protein, gliadin, glutenin, glutenin macropolymers (GMP), high- and low-molecular weight glutenin subunits (HMW-GS and LMW-GS), and the ratio of gliadin to glutenin in wheat flour

Treatment	Protein (%)	Gliadin (%)	Glutenin (%)	Gliadin/ Glutenin	GMP (%)	HMW-GS (%)	LMW-GS (%)
TL5	13.85^c^	3.14^c^	3.36^c^	0.93^a^	3.18^c^	0.86^b^	2.54^b^
TL3	14.38^b^	3.59^b^	4.04^b^	0.89^a^	3.97^b^	0.90^b^	2.68^b^
TL1	15.79^a^	4.45^a^	5.00^a^	0.89^a^	4.96^a^	1.01^a^	3.18^a^

TL5, TL3 and TL1 indicate topdressing timing of nitrogen at the growth stage of fifth, third and first leaf (flag leaf) from the top, respectively. Letters indicate significant differences at *P* < 0.05

In accordance with the protein content, amino acid contents in flour were also modified by N topdressing timing as shown in Additional file 1: Figure S1. Glutamic acid, which is the principal component of protein in flour, occupies more than 50% of total amino acids. The content of Glu was 19.9% higher in TL1 than in TL3. The contents of Leu, Phe and Val were also higher in TL1 but lower in TL5 in comparison with TL3 (Additional file 1: Figure S1).

## Discussion

Grain protein content is one of the major breeding objectives in wheat due to its essential role for determining the rheological qualities of flour [27]. The genetic background and the environment, as well as their interaction, play a role in determining the protein content and composition. Among environmental factors, nitrogen availability during wheat development is one of the most important parameters determining the gluten protein polymer structure [11]. In addition, split application of N fertilizer in combination with delayed timing of application of N by topdressing is a very important agronomic practice both to achieve high grain yield and protein content and to realize economic and ecological production of wheat by reducing N fertilizer dose [13]. Here, in a field experiment the three topdressing N timing treatments were designed according to the leaf stage, i.e. N was applied at the emergence of the top 5th (TL5), 3rd (TL3) and 1st leaf (TL1) of the main stem, respectively. TL3 was regarded as the control since this stage is the jointing stage, which is the recommendation application stage for applying N topdressing. In agreement with previous reports [28, 29], delaying N topdressing (TL1) increased the contents of protein, gliadin and glutenin (Table 1. Contents of gliadin and glutenin showed synchronous increases in TL1, resulting in an unaltered gliadin to glutenin ratio (Table 1). Xue et al. (2016) also observed that splitting and delaying basal N topdressing to the late booting or heading stage enhanced the percentages of gliadin and glutenin without changing the ratio of gliadin to glutenin [28]. However, in another study, gliadin was reported to be more easily modified than glutenin by late N application, resulting in an increased ratio of gliadin to glutenin [16, 17]. The divergence with the current study may be due to differences in genotype and environmental factors, which have been reported to play important roles in regulating protein quality [11, 16, 17].

The N accumulated in vegetative organs before anthesis and which is redistributed into grain after anthesis, accounts for more than 75% of the total N in final dry wheat grain, while N absorbed and directly transported to grain after anthesis accounts for less than 25% [30]. Thus, the pre-anthesis accumulated N is crucial for accumulation of storage proteins in wheat grain, and to a large extent, determines the processing quality of wheat flour [31]. GS is known to play an important role in the assimilation of mineral N in plants [32]. Here, the expression in flag leaves of the encoding gene *GS2a* substantially decreased during grain filling (Fig. 1), indicating declining protein synthesis in the flag leaf. However, delayed N topdressing (TL1) upregulated the expression of *GS2a* in flag leaves (compared with normal timing of topdressing application, TL3), while early N application (TL5) had the opposite effect. In summary these observations indicate that delaying the timing of N topdressing application improves both the uptake of N in the vegetative phase, but it also increases the physiological function of protein degradation and amino acid transport from leaves to grains during grain filling. Together with GS, asparagine synthetase (AS), which catalyses the formation of asparagine (Asn) and glutamate (Glu) from aspartate and glutamine (Gln), is believed to play a crucial role in primary N metabolism [33]. As Asn can be used for long-range transport and storage compounds and has a higher N:C ratio than Gln, it is vital for N assimilation [34]. The expression of *ASN1* was significantly higher in treatments with late topdressing (TL1) after 13DAA (Fig. 1). This is inconsistent with a previous report that a higher expression of *TaASN1* did not result in higher N use efficiency [24]. The difference with our observation may be due to different sampling time and because the previous work focused only on the expression between tillering stage to anthesis while the focus of this work was the period after anthesis. AlaAT catalyses the production of L-alanine (Ala) and 2-oxoglutarate from pyruvate and L-glutamate (Glu). Transgenic lines with overexpression of AlaAT have a significantly higher level of key N assimilation metabolites in their shoots, such as Gln, Glu and Arg [35]. In general, higher expression of *GS2a*, *ASN*, *AlaAT* in TL1 indicated a higher N assimilation in leaves.

For a more detailed insight into the contribution of vegetative N reserves to the developing grain, we studied the proteolytic activity in leaves. The most important source of N in leaves is the soluble proteins, such as Rubisco, which accounts for more than 50% of total leaf cell protein [36]. Therefore proteolysis of soluble proteins in leaves, which is catalyzed by various proteolytic enzymes, is essential for supply of amino acids for protein synthesis in grain [12]. Cysteine protease are the most abundant group of protease responsible for such degradation and mobilization of proteins during grain filling [37]. Here, the expression of genes encoding thiol protease and cysteine protease was higher in TL1 (Fig. 1), indicating a stronger degradation and mobilization of storage protein in leaf with delayed N topdressing. At 31 DAA, TL1 had a remarkable higher expression level than TL3 of both protease genes, reflecting a potential by late topdressing application for acceleration of senescence and subsequent pleiotropic effects on N remobilization and total GPC [38]. PPDK catalyzes the interconversion between pyruvate and phosphoenolpyruvate, which is an important step in the synthesis of the transported amino acid glutamine. Therefore *PPDK* gene regulation plays an important role for N remobilization from leaves during both dark-induced and age-dependent senescence [39]. In agreement with this role we found that the expression of *PPDK* was higher during middle and late filling stages in TL1 (Fig. 1), contributing to the apparent higher amino acids remobilization to grains in delayed topdressing application.

During grain filling, the amino acids produced by proteolysis in leaves are exported via the phloem [40] to the sink tissues as substrate for protein synthesis [41]. In developing wheat grains, phloem assimilates are first unloaded into the apoplastic endosperm cavity and then imported into endosperm by transfer cells [42]. Most likely the amount of exported amino acids into phloem sap does not depend on the content of the total amino acids in leaf [12], since the rate of amino acid exportation is dependent on the N available to the plant. In addition, the composition of amino acids does not change during the transportation in the sieve tubes leading to the endosperm cavity [41]. Therefore, the composition and level of amino acids in both endosperm and endosperm cavity sap in response to N application were directly concerned in this study.

The endosperm cavity sap has no detectable metabolic activity in itself, indicating that the endosperm cavity functions mostly as a conveyer of nutrients for developing grains [41]. In addition, it also serves as temporary storage for accumulating reserves that are mobilized during early seed filling. In agreement with this, we observed that the content of free amino acids in the cavity sap dropped sharply from 7 to 13 DAA, suggesting a rapid transportation of free amino acids from the cavity to the actual endosperm. This is in line with the suggested timing of completion of endosperm differentiation and the beginning of storage product accumulation between 7 and 9 DAA [42]. We observed a significant difference between treatments in the levels of free amino acids at 7 DAA and 13 DAA in cavity sap, while very little variation was observed at 25 DAA (Fig. 2a). This suggested that the levels of free amino acids in endosperm cavity sap in plants with delayed topdressing (TL1) provided more stored amino acids for the subsequent protein synthesis in the endosperm.

In endosperm, the content of total free amino acids decreased steadily during grain filling, and finally with a more rapid decrease from 19 to 31 DAA (Fig. 2b), which corresponds to the most active storage synthesis stage in grain filling [43]. Delaying N topdressing increased the content of total free amino acids in the endosperm at 7 DAA and 13 DAA, while there was no difference between topdressing timing treatments at 31 DAA (Fig. 2). This observation is well in accordance with a previous report saying that at 7 and 14 DAA, the concentration of total free amino acids in grains was lower in plants with low N treatment that resulted in lower grain protein content in comparison to that of high N treatment. Still in those plants no differences were observed between free grain amino acid concentrations after 21 DAA [44]. This suggested that at early filling stages more free amino acids are utilized for synthesis of protein in grains in TL1 than in the other topdressing treatments.

Amino acid composition is different between plant species; however, there is a consensus that Gln is the dominating amino acid of phloem sap (similar to endosperm cavity sap) in barley, wheat and spinach [45–47]. For technical reasons we measured only the pool of Glu and Gln in this study. However, we confirmed that Glu and Gln were the most abundant amino acids in both cavity sap (Fig. 3a) and endosperm (Additional file 1: Table S4–2), reflecting the role of Gln as a major transported amino acid. After uptake from the endosperm cavity into endosperm, the content of Glu seems to decrease rapidly from 7 to 19 DAA in endosperm, while the content of Ser, Pro, Gly, Leu and Thr increases, suggesting that these amino acids are synthesized in the endosperm during this period, utilizing Glu delivered to the grain. This observation is in accordance with previous report [44].

Since glutenin of mature grain mainly consist of Gln, Gly, Pro, Ser, Tyr, Leu and Ala, which constitute more than 75% of glutenins total amino acids [48], conversions between amino acids are crucial to provide appropriate substrate for glutenin synthesis. Levels of accumulation of different types of seed storage proteins have been reported to be influenced by differences in the interconversion between amino acids [49]. The expression of genes encoding pyrroline-5-carboxylate reductase, arogenate dehydrogenase, phosphoserine aminotransferase, isopropyl malate dehydrogenase, alanine aminotransferase and alanine glyoxylate aminotransferase, which catalyze the formation of Gly, Pro, Ser, Tyr, Leu and Ala, respectively [44], were studied here. Overall, the expression of these genes during the grain filling stage were higher in TL1 (Fig. 3b), indicating that the conversion of amino acids during grain filling is more active in TL1 than in the other two treatments. Such higher activity is likely to benefit the glutenin biosynthesis in grain.

The expression of genes coding HMW-GS and LMW-GS was highest at 25 DAA for all three topdressing timings. Also, the differences between treatments were also most significant at this stage (Fig. 4). This is in accordance with previous studies showing that the differences in content of HMW-GS due to different N application regimes in wheat starts to occur at 21 DAA and gradually becomes more prominent from 28 DAA to 35 DAA [22]. Formation of disulfide bonds, which is a prerequisite for correct folding of seed storage proteins is carried out by PDI-like proteins that are therefore important for gluten quality and content of GMP [25]. Here we found that the expression of *PDIL2–1* was higher in TL1, in line with the observation that the content of GMP was increased by delaying N topdressing.

## Conclusion

**Fig. 5:**
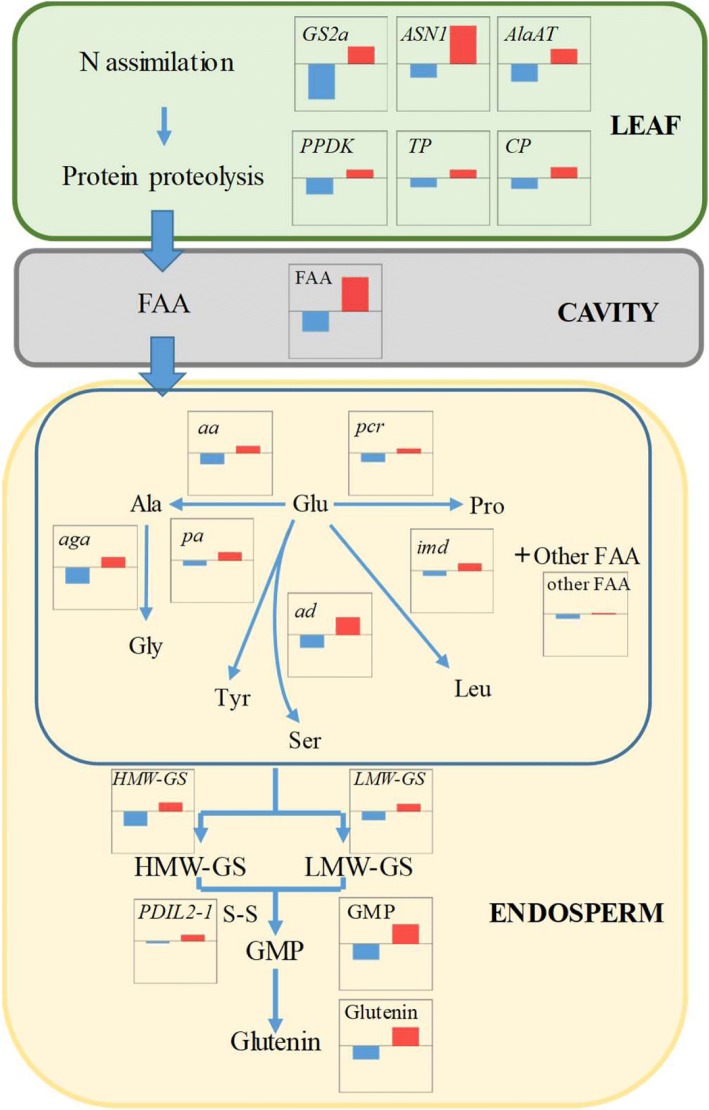
Schematic presentation of N roadmap for grain gluten quality. Note: The main protein degradation is carry out in flag leaves, resulting in production of free amino acids that are transported from the leaf to the endosperm cavity sap, and subsequently used for protein synthesis in the wheat grain. This transportation flux is indicated with blue arrows. TL3 was used as standard. The bars indicate the relative levels of (TL1, red) and (TL5, blue) of: expression levels of relevant genes for amino acid metabolism, amino acid concentrations and protein content. TL5, TL3 and TL1 indicate topdressing timing of nitrogen in different wheat leaf fifth, third and first leaf (flag leaf) from the top, respectively. The average gene expression of selected genes in leaf (a-f) and endosperm (h-m, o-q) during grain filling stage was used to calculate the relative gene expression change in TL5 and TL1 in comparison to TL3. Total content of free amino acids in the cavity sap at 7 DAA were compared between treatments. The relative changes of average content of other free amino acids in endosperm during the grain filling stage in TL5 and TL1 are shown. The scaling range of vertical axis in all the bar charts is from − 0.5 to 0.5. a, *GS2a; b, ASN1; c, AlaAT; d, PPDK; e, Thiol protease; f, Cysteine protease; g,* FAA content*; h, pyrroline-5-carboxylate reductase*; i, *phosphoserine aminotransferase*; j, *arogenate dehydrogenase*; k, *isopropyl malate dehydrogenase*; l, *alanine aminotransferase*; m, *alanine glyoxylate aminotransferase*; n, other FAA content; o, *HMW-GS*; p, *LMW-GS*; q, *PDIL2–1*; r, GMP content; s, Glutenin content. FAA, free amino acid

In summary, we have defined a schematic roadmap showing the coordinated regulation of N assimilation, source tissue protein proteolysis, amino acid transportation and metabolism as well as storage protein accumulation (Fig. 5). The results presented here suggest that there is a better coordination of N assimilation and leaf protein proteolysis when delaying N topdressing (TL1), which provides continuous N reserves for transportation to grains in the filling stage. While there were distinct differences in content of free amino acids in the endosperm cavity sap at 7 DAA, very little variation between topdressing treatments was observed at late grain filling stage, confirming N remobilization capacity was enhanced by TL1. Furthermore, the combined analysis of amino acids metabolism and glutenin synthesis in endosperm revealed an improved protein production in grain throughout development in grains from plants with delayed N topdressing. As an expected result of this, the contents of H/LMW-GS, GMP and glutenin were increased in TL1. The result provides a detailed understanding of the relationship between N remobilization, final grain protein content, gluten quality and timing of N topdressing in bread wheat.

## Additional file

Additional file 1: **Table S1.** Function of genes involving in nitrogen metabolism, protease synthesis and glutenin synthesis. **Table S2.** List of primers for qRT-PCR analysis of genes. **Table S3.** Nitrogen content in wheat plants and rate of nitrogen transported from vegetative organs to grains. **Table S4–1.** Contents of free amino acids in sap from wheat grain cavity. **Table S4–2.** Contents of free amino acids in endosperm. **Figure S1.** Effects of nitrogen topdressing timing on contents of amino acids in flour. (PDF 235 kb)

## Abbreviations

*aa*: *Alanine aminotransferase*
*ad*: *Arogenate dehydrogenase*
ADP-RF: ADP-ribosylation factor
*aga*: *Alanine glyoxylate aminotransferase*
Ala: Alanine
AlaAT: Alanine aminotransferase
ANOVA: Analysis of variance
Arg: Arginine
AS: Asparagine synthetase
Asp: Aspartate
Cys: Cysteine
DAA: Days after anthesis
Gln: Glutamine
Glu: Glutamate
Gly: Glycine
GMP: Glutenin macropolymers
GS: Glutamine synthetase
His: Histidine
HMW-GS: High molecular weight glutenin subunits
*imd*: *Isopropyl malate dehydrogenase*
Leu: Leucine
LMW-GS: Low molecular weight glutenin subuni
LSD: Least significant difference
Lys: Lysine
Met: Methionine
N: Nitrogen
*pa*: *Phosphoserine aminotransferase*
*pcr*: *Pyrroline-5-carboxylate reductase*
PPDK: Cytosolic pyruvate orthophosphate dikinase
Pro: Proline
qRT-PCR: Quantitative real-time PCR
SDS: Sodium dodecyl sulfate
Ser: Serine
Trp: Tryptophan
Val: Valine
