# Supplementary material for: Nitrogen topdressing timing modifies free amino acids profiles and storage protein gene expression in wheat grain
Source: BMC Plant Biol. 2018 Dec 13;18:353. doi: 10.1186/s12870-018-1563-3 (PMC6293556; doi:10.1186/s12870-018-1563-3)
Supplement: Supplementary file 1 — Table S1. Function of genes involving in nitrogen metabolism, protease synthesis and glutenin synthesis. Table S2. List of primers for qRT-PCR analysis of genes. Table S3. Nitrogen content in wheat plants and rate of nitrogen transported from vegetative organs to grains. Table S4–1. Contents of free amino acids in sap from wheat grain cavity. Table S4–2. Contents of free amino acids in endosperm. Figure S1. Effects of nitrogen topdressing timing on contents of amino acids in flour. (PDF 235 kb) [file 12870_2018_1563_MOESM1_ESM.pdf]

Table S1 Function of genes involving in nitrogen metabolisms, protease synthesis and glutenin synthesis

gene	function	reference
GS2a	Glutamine synthetase catalyzing ammonium to incorporate into organic molecules.	(Good et al., 2004)
PPDK	Cytosolic pyruvate orthophosphate dikinase catalyzing the reversible conversion of pyruvate to phosphoenolpyruvate.	(Cooper and Kornberg, 1967)
A1aAT	Alanine aminotransferase catalyzing the synthesis of alanine and 2-oxoglutarate from pyruvate and glutamate.	(Good et al., 2007)
ASN1	Asparagine synthetase catalysing the formation of asparagine and glutamate from glutamine and aspartate.	(Good et al., 2004)
cysteine protease	Involving of reorganization of plant metabolism, remodeling of cell protein components, degradation of damaged or unnecessary proteins and nutrient remobilization.	(M. Grudkowska et al.,2004)
PDIL	Possessing a known function of assisting disulfide bond formation.	(Wang et al, 2013)
HMW-x(y)	Encoding high molecular weight glutenin subunits	-
LMW-A(B)(D)	Encoding low molecular weight glutenin subunits	-

Table S2 List of primers for qRT-PCR analysis of genes

Gene	Forward（5’ → 3’）	Reverse（5’ → 3’）
GS2a	GGAACCCGTCCCTACTAAC	GTCTCCCGCATCAATACC
PPDK	GACCAACCAGGGTATTCA	TAGTCCCAGCTTTGTAATC
AlaAT	GCAACAAGGCTGAGGGTG	TCCGCTTTCAATCGTAGTG
ASN1	GAGCATCTCCCAGCAACCATCATG	GGCAAGCAGGACAGGACACCATCAAC
cysteine protease	GCCGGAGAAGAGTACTGGAT	TACATGACGGGGTAGGCAC
thiol protease	CTGGTTTGTACGACGGCATG	AGACGTAGCCCTTCTTTCCC
Pyrroline-5-carboxylate reductase	AAGCCCCAGATTGTGAAGCA	TGACCAGACCAATCCTGCAA
Arogenate dehydrogenase	ACTCCAATGCCAACCCAGAT	CCATCCGGGGAAAGAAAAGC
Phosphoserine aminotransferase	GGTAATACGAAGAGGCCGGA	GCAATTTACCGGTCACCTGG
Isopropyl malate dehydrogenase	TTGGGTGCAGGAATTTAGCG	CAGCCCAGGTGCAAAGGG
Alanine aminotransferase	GCTAACGATGCAAGGATCCC	TTCCAATATCACCGGCCAGA
Alanine glyoxylate aminotransferase	TGTACGACGAACAAGGCAAG	ATATGGTGGTGGTGTGCTGA
HMW-x	ATGTTAGCGCGGAGCACCAG	CTATCACTGGCTGGCCGACA
HMW-y	CAAGGCTACGACAGCCCATAC	CACCCTCCATCCGACACACT
LMW-A	TCAGCARCAACAACCCCAAC	TGTTGTTGTTGAGGYTGTTG
LMW-B	GGTACCTTDTTGCAGCCACA	CCGAATGGCACAMTAGTGGT
LMW-D	TTGCAGCCACACCARATAGC	CTTATCAGTAGGCACCAACT
PDIL2-1	TCCCCACGATACTCTTCTATCCA	CGGTCCCCCTCGAAAGTTAT

Table S3 Nitrogen content in wheat plants and rate of nitrogen transported from vegetative organs to grains

Treatment	Nitrogen content in single stem (mg)							Nitrogen transport rate (%)
	Anthesis			Mature				
	Stem	Leaf	Glume	Stem	Leaf	Glume	Grain	
TL5	9.92[b]	24.61[c]	5.86[a]	3.63[a]	0.59[b]	1.25[a]	55.57[b]	62.89[c]
TL3	14.76[a]	27.26[b]	6.11[a]	1.60[b]	1.41[a]	0.62[b]	67.23[a]	66.19[b]
TL1	14.73[a]	29.67[a]	7.06[a]	3.28[a]	0.92[b]	0.38[b]	66.20[a]	70.8[a]

Note: TL5, TL3 and TL1 indicate topdressing timing of nitrogen at the growth stage of fifth, third and first leaf (flag leaf) from the top, respectively. Different small letters in the same column of each material are significantly different at the 0.05 probability level.

Table S4-1 Contents of free amino acids in sap of the endosperm cavity in wheat

Time	Treatment	Amino acid (arbitrary unit)																
		Glu	Ser	Ala	Lys	Val	Leu	Pro	Ile	Arg	Phe	Met	Thr	His	Asp	Gly	Cys	Tyr
7DAA	TL5	29.48^{c}	7.84^{c}	5.24^{c}	2.96^{c}	3.09^{b}	2.62^{b}	2.74^{c}	1.05^{c}	2.00^{b}	2.66^{c}	1.82^{b}	1.83^{c}	2.26^{b}	0.76^{c}	0.60^{b}	0.91^{a}	1.44^{a}
	TL3	41.58^{b}	10.77^{b}	7.58^{b}	4.34^{b}	3.75^{b}	3.09^{b}	3.15^{b}	1.76^{b}	2.26^{b}	3.03^{b}	2.13^{b}	2.62^{b}	2.44^{b}	1.15^{b}	0.82^{b}	1.07^{a}	1.12^{a}
	TL1	51.98^{a}	16.76^{a}	10.22^{a}	8.36^{a}	6.18^{a}	4.94^{a}	4.90^{a}	4.81^{a}	4.11^{a}	3.87^{a}	3.34^{a}	3.15^{a}	3.02^{a}	1.92^{a}	1.67^{a}	1.49^{a}	1.45^{a}
13DAA	TL5	8.88^{c}	9.24^{b}	5.98^{c}	7.20^{b}	2.09^{b}	1.95^{b}	1.57^{b}	0^{b}	0.97^{b}	0.68^{b}	1.83^{a}	0.83^{b}	0.56^{b}	0.19^{c}	2.21^{b}	1.19^{a}	0.85^{a}
	TL3	13.67^{b}	10.22^{b}	9.58^{b}	9.99^{a}	2.53^{a}	2.29^{b}	2.10^{a}	0^{b}	1.02^{b}	0.98^{a}	1.98^{a}	1.11^{b}	0.91^{a}	0.67^{b}	2.84^{a}	1.32^{a}	1.00^{a}
	TL1	17.77^{a}	13.15^{a}	12.06^{a}	11.00^{a}	2.54^{a}	2.80^{a}	2.16^{a}	1.46^{a}	1.22^{a}	1.11^{a}	2.08^{a}	1.49^{a}	0.87^{a}	1.21^{a}	3.02^{a}	1.61^{a}	1.08^{a}
19DAA	TL5	4.65^{c}	9.42^{a}	5.13^{c}	8.44^{b}	1.06^{b}	1.65^{b}	0.55^{b}	0^{a}	0.78^{b}	0.17^{c}	0.83^{a}	0.58^{a}	0.51^{b}	0.26^{b}	1.91^{c}	1.53^{b}	0^{a}
	TL3	8.59^{b}	9.87^{a}	7.75^{b}	9.80^{b}	1.06^{b}	1.48^{b}	0.63^{b}	0^{a}	0.85^{b}	0.24^{b}	0.87^{a}	0.39^{b}	0.51^{b}	0.45^{a}	2.45^{b}	1.40^{b}	0^{a}
	TL1	13.20^{a}	9.89^{a}	9.91^{a}	14.97^{a}	2.16^{a}	2.39^{a}	0.92^{a}	0^{a}	1.04^{a}	0.4^{a}	0.85^{a}	0.72^{a}	1.01^{a}	0.42^{a}	2.83^{a}	2.44^{a}	0^{a}
25DAA	TL5	4.17^{a}	6.02^{a}	2.78^{b}	4.51^{a}	0.80^{b}	0.83^{c}	0.24^{c}	0^{a}	0.50^{b}	0^{b}	0.64^{b}	0.47^{a}	0.32^{b}	0^{b}	0.61^{a}	1.02^{a}	0^{a}
	TL3	5.82^{a}	5.57^{a}	2.47^{b}	4.18^{a}	0.96^{b}	1.22^{b}	0.45^{b}	0^{a}	0.83^{b}	0.05^{a}	0.72^{b}	0.44^{a}	0.42^{b}	0.17^{a}	0.51^{a}	1.19^{a}	0^{a}
	TL1	6.16^{a}	6.00^{a}	3.08^{a}	4.61^{a}	1.33^{a}	1.67^{a}	0.71^{a}	0^{a}	1.23^{a}	0.11^{a}	0.92^{a}	0.49^{a}	0.73^{a}	0.16^{a}	0.81^{a}	1.43^{a}	0^{a}
Source of variation	d.f.	ANOVA P values																
Treatment	2	8	0.21	2.4	0.67	n.s.	n.s.	4.18	1.81	7.59	8.76	n.s.	n.s.	n.s.	3.12	7.81	n.s.	n.s.
Time	3	7.11	116.5	65.96	77.4	27.29	226.3	379.1	86.83	118.7	33.21	104.1	12.81	38.76	18.27	32.01	0.32	12.81

Note: TL5, TL3 and TL1 indicate topdressing timing of nitrogen at the growth stage of fifth, third and first leaf (flag leaf) from the top, respectively. The content of the free amino acid in endosperm sap basing on thirty grains was expressed on an arbitrary unit because of the difficulty to accurately assay the quantity of the sap. All data were subjected to one-way analysis of variance (ANOVA) to determine the significant differences between treatments or time independently. Different small letters in the same column are significantly different at the 0.05 probability level in consideration of treatment only. d.f., degrees of freedom; n.s., not significant (P >0.05).

Table S4-2 Contents of free amino acids in endosperm

Time	Treat-ment	Amino acid (mg g^{-1} dwt)																
		Glu	Ser	Pro	Gly	Val	Leu	Ala	Tyr	Lys	Ile	Arg	Phe	Met	Thr	His	Asp	Cys
7DAA	TL5	3.29^{c}	1.28^{b}	0.61^{b}	1.10^{b}	0.73^{c}	0.93^{a}	0.82^{a}	0.51^{a}	0.65^{a}	0.56^{a}	0.34^{b}	0.28^{a}	0.34^{a}	0.38^{a}	0.21^{a}	0.12^{a}	0.02^{a}
	TL3	3.61^{b}	1.66^{a}	0.63^{b}	1.19^{ab}	0.87^{b}	0.92^{a}	0.85^{a}	0.57^{a}	0.62^{a}	0.55^{a}	0.35^{b}	0.31^{a}	0.38^{a}	0.36^{a}	0.20^{a}	0.13^{a}	0^{b}
	TL1	3.90^{a}	1.66^{a}	1.16^{a}	1.27^{a}	1.00^{a}	0.89^{a}	0.80^{a}	0.59^{a}	0.56^{a}	0.48^{a}	0.42^{a}	0.34^{a}	0.32^{a}	0.32^{a}	0.18^{a}	0.13^{a}	0^{b}
13DAA	TL5	0.99^{b}	1.91^{b}	0.90^{c}	1.29^{c}	0.44^{a}	0.17^{a}	0.54^{a}	0.72^{a}	0.29^{a}	0.38^{a}	0.27^{a}	0.17^{a}	0.49^{b}	0.23^{a}	0.10^{a}	0.15^{a}	0.09^{a}
	TL3	1.07^{b}	2.06^{b}	1.13^{b}	1.40^{b}	0.45^{a}	0.21^{a}	0.60^{a}	0.73^{a}	0.32^{a}	0.41^{a}	0.27^{a}	0.19^{a}	0.58^{ab}	0.27^{a}	0.09^{a}	0.15^{a}	0.01^{b}
	TL1	1.37^{a}	2.54^{a}	1.49^{a}	2.04^{a}	0.56^{a}	0.22^{a}	0.57^{a}	0.72^{a}	0.32^{a}	0.43^{a}	0.24^{a}	0.23^{a}	0.63^{a}	0.28^{a}	0.10^{a}	0.16^{a}	0.01^{b}
19DAA	TL5	0.46^{a}	1.53^{b}	1.88^{c}	0.91^{b}	0^{a}	0.34^{b}	0.34^{a}	0.37^{b}	0.17^{b}	0.17^{a}	0.21^{a}	0.14^{a}	0.43^{b}	0.25^{b}	0.08^{a}	0.21^{a}	0.01^{a}
	TL3	0.52^{a}	1.52^{b}	2.16^{b}	1.09^{b}	0^{a}	0.41^{b}	0.44^{a}	0.43^{ab}	0.23^{a}	0.21^{a}	0.25^{a}	0.13^{a}	0.56^{a}	0.32^{ab}	0.09^{a}	0.22^{a}	0.01^{a}
	TL1	0.44^{a}	2.12^{a}	2.33^{a}	1.44^{a}	0^{a}	0.56^{a}	0.4^{a}	0.44^{a}	0.23^{a}	0.17^{a}	0.22^{a}	0.17^{a}	0.57^{a}	0.37^{a}	0.09^{a}	0.22^{a}	0.06^{a}
25DAA	TL5	0.13^{b}	0.86^{b}	1.12^{c}	0.54^{a}	0^{a}	0.26^{a}	0.14^{a}	0.21^{a}	0.06^{a}	0.15^{a}	0.12^{a}	0.08^{a}	0.17^{a}	0.09^{a}	0.05^{a}	0.04^{a}	0^{b}
	TL3	0.13^{b}	1.01^{a}	1.42^{b}	0.59^{a}	0^{a}	0.28^{a}	0.19^{a}	0.26^{a}	0.08^{a}	0.16^{a}	0.19^{a}	0.07^{a}	0.18^{a}	0.13^{a}	0.07^{a}	0.04^{a}	0.12^{a}
	TL1	0.62^{a}	1.03^{a}	1.71^{a}	0.57^{a}	0^{a}	0.28^{a}	0.20^{a}	0.30^{a}	0.07^{a}	0.16^{a}	0.21^{a}	0.11^{a}	0.19^{a}	0.14^{a}	0.08^{a}	0.04^{a}	0.11^{a}
31DAA	TL5	0.09^{a}	0.22^{a}	0.17^{a}	0.13^{a}	0^{a}	0.11^{a}	0.01^{a}	0.05^{a}	0.03^{a}	0^{a}	0.02^{a}	0.01^{a}	0.06^{a}	0.05^{a}	0.04^{a}	0.05^{a}	0.04^{a}
	TL3	0.11^{a}	0.29^{a}	0.19^{a}	0.15^{a}	0^{a}	0.12^{a}	0.06^{a}	0.06^{a}	0.05^{a}	0^{a}	0.04^{a}	0.01^{a}	0.08^{a}	0.06^{a}	0.04^{a}	0.03^{a}	0.02^{a}
	TL1	0.12^{a}	0.30^{a}	0.20^{a}	0.16^{a}	0^{a}	0.13^{a}	0.06^{a}	0.07^{a}	0.06^{a}	0^{a}	0.04^{a}	0.01^{a}	0.08^{a}	0.07^{a}	0.05^{a}	0.04^{a}	0.04^{a}
Mature	TL5	0.13^{a}	0.26^{a}	0^{a}	0^{a}	0^{a}	0^{a}	0^{a}	0^{a}	0^{a}	0^{a}	0.18^{a}	0.10^{a}	0^{a}	0^{a}	0.03^{a}	0^{a}	0.14^{a}
	TL3	0.13^{a}	0.10^{b}	0^{a}	0^{a}	0^{a}	0^{a}	0^{a}	0^{a}	0^{a}	0^{a}	0.16^{a}	0.09^{a}	0^{a}	0^{a}	0.02^{a}	0^{a}	0.13^{a}
	TL1	0.09^{a}	0.05^{b}	0^{a}	0^{a}	0^{a}	0^{a}	0^{a}	0^{a}	0^{a}	0^{a}	0.10^{a}	0.08^{a}	0^{a}	0^{a}	0.02^{a}	0^{a}	0.10^{a}
Source of variation	d.f.	ANOVA P values																
Treatment	2	7.95	1.54	3.51	1.83	n.s.	n.s.	n.s.	n.s.	3.11	n.s.	n.s.	n.s.	n.s.	n.s.	n.s.	n.s.	n.s.
Time	5	174.3	399.4	287.7	331.2	67.21	325.7	233.2	197.4	130.2	60.96	182.0	23.37	93.64	17.12	117.6	5.17	229.2

Note: TL5, TL3 and TL1 indicate topdressing timing of nitrogen at the growth stage of fifth, third and first leaf (flag leaf) from the top, respectively. All data were subjected to one-way analysis of variance (ANOVA) to determine the significant differences between treatments or time independently. Different small letters in the same column are significantly different at the 0.05 probability level in consideration of treatment only. d.f., degrees of freedom; n.s., not significant ($P > 0.05$).

Fig. S1 Effects of nitrogen topdressing timing on contents of amino acids in wheat flour

Note: TL5, TL3 and TL1 indicate topdressing timing of nitrogen at the growth stage of fifth, third and first leaf (flag leaf) from the top, respectively. Bars indicate standard errors.

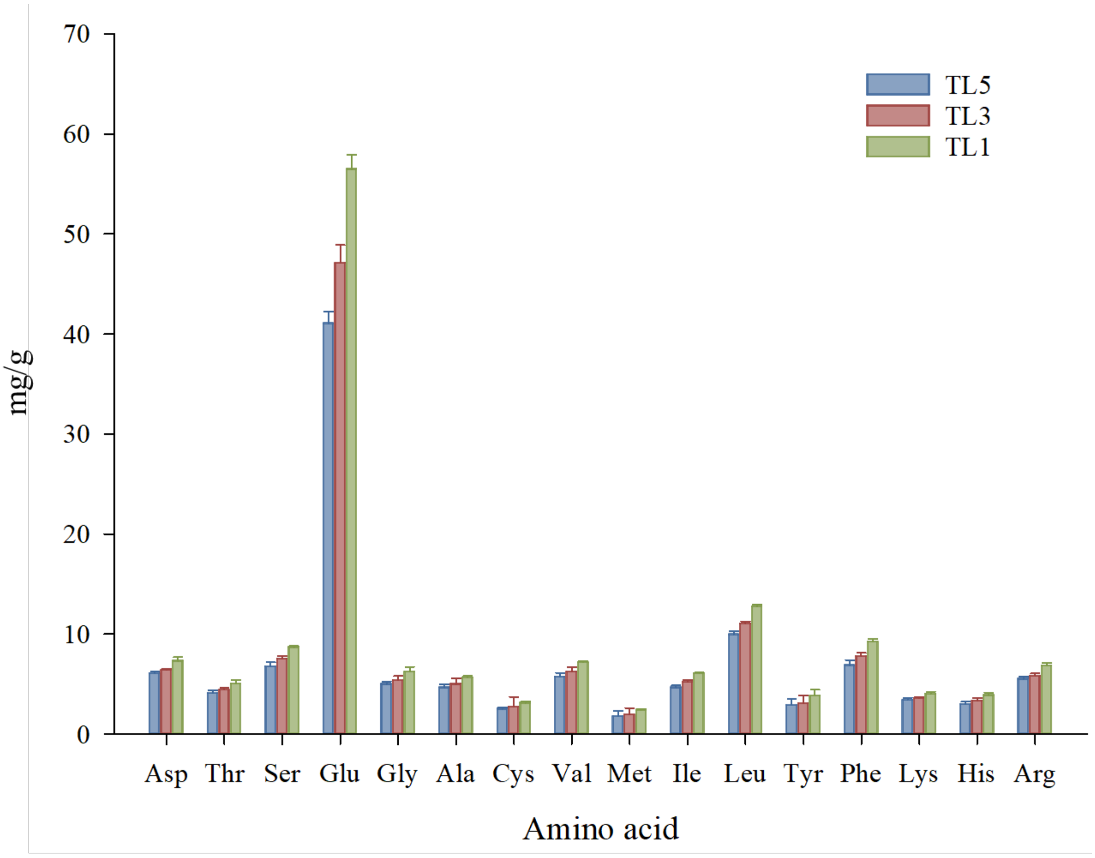
70
60
50
40
30
20
10
0
mg/g
TL5
TL3
TL1
Asp Thr Ser Glu Gly Ala Cys Val Met Ile Leu Tyr Phe Lys His Arg
Amino acid

Fig. S1
